# Gut-Induced Inflammation during Development May Compromise the Blood-Brain Barrier and Predispose to Autism Spectrum Disorder

**DOI:** 10.3390/jcm10010027

**Published:** 2020-12-24

**Authors:** Rebecca S. Eshraghi, Camron Davies, Rahul Iyengar, Linda Perez, Rahul Mittal, Adrien A. Eshraghi

**Affiliations:** 1 Hearing Research and Communication Disorders Laboratory, Department of Otolaryngology, University of Miami Miller School of Medicine, Miami, FL 33136, USA; cdavi191@med.fiu.edu (C.D.); rsi21@med.miami.edu (R.I.); dralindaperez18@gmail.com (L.P.); r.mittal11@med.miami.edu (R.M.); aeshraghi@med.miami.edu (A.A.E.); 2 Department of Neurological Surgery, University of Miami Miller School of Medicine, Miami, FL 33136, USA; 3 Department of Biomedical Engineering, University of Miami, Coral Gables, FL 33146, USA; 4 Department of Pediatrics, University of Miami Miller School of Medicine, Miami, FL 33136, USA

**Keywords:** gut-brain axis, gut microbiota, autism spectrum disorder, immunological mediators, gastrointestinal problems, neuroinflammation, short chain fatty acids, blood-brain barrier, inflammation

## Abstract

Recently, the gut microbiome has gained considerable interest as one of the major contributors to the pathogenesis of multi-system inflammatory disorders. Several studies have suggested that the gut microbiota plays a role in modulating complex signaling pathways, predominantly via the bidirectional gut-brain-axis (GBA). Subsequent in vivo studies have demonstrated the direct role of altered gut microbes and metabolites in the progression of neurodevelopmental diseases. This review will discuss the most recent advancements in our understanding of the gut microbiome’s clinical significance in regulating blood-brain barrier (BBB) integrity, immunological function, and neurobiological development. In particular, we address the potentially causal role of GBA dysregulation in the pathophysiology of autism spectrum disorder (ASD) through compromising the BBB and immunological abnormalities. A thorough understanding of the complex signaling interactions between gut microbes, metabolites, neural development, immune mediators, and neurobiological functionality will facilitate the development of targeted therapeutic modalities to better understand, prevent, and treat ASD.

## 1. Introduction

Autism spectrum disorders (ASDs) are characterized by a multifaceted range of neurobiological disorders with functional deficits in both social communication and cognitive domains as well as behavior abnormalities [1]. While numerous genetic and environmental factors are implicated in the complex pathophysiology of ASD and abnormal behavior manifestations, recent investigations have suggested that there may be a causal role of the gut microbiome [2,3].

**Figure 1:**
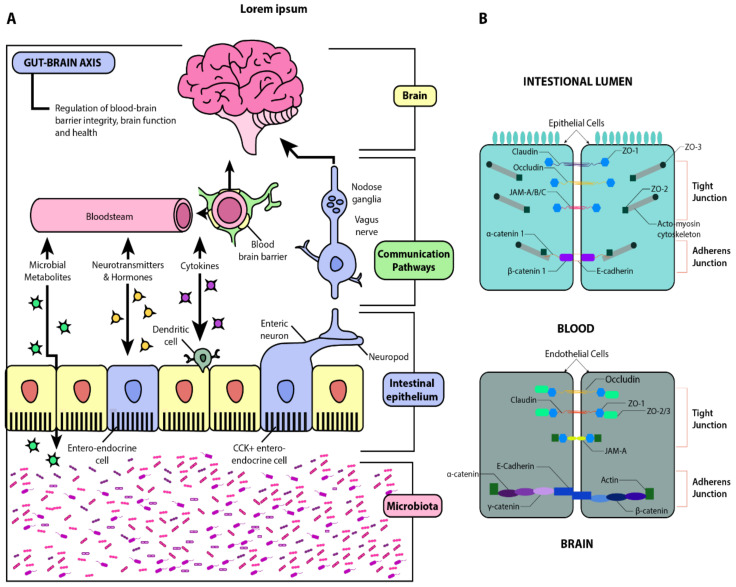
Schematic illustration of the gut-brain axis (GBA). Panel (**A**): Beginning with the microbiota, this diagram illustrates the interactions between the gut and the brain via microbial metabolites, cytokines, neurotransmitters, and hormones. The gut-brain humoral connections which intersect at the blood-brain barrier and the neuronal connections by way of the enteric nervous system and vagus nerve are also illustrated. Through these pathways, dysbiosis and increased barrier permeability may affect neural development, maturation, and function. Panel (**B**): Structural similarities between the blood-brain barrier (BBB) and the intestinal epithelial barrier. Adapted from [5,6].

The microbiome bi-directionally modulates the gut-brain-axis (GBA) through complex neuroendocrine, neuro-immune, and autonomic nervous signaling mechanisms [4] (Figure 1).

Furthermore, emerging studies have suggested an association between impaired gut microbiota and disrupted neurobiological development [4,5,6,7,8]. Clinical investigations of gut dysbiosis indicate a high coincidence of gastrointestinal (GI) symptoms and compositional changes within the gut microbiome in individuals with ASD [7,8]. Subsequent studies suggest that the degree of GI symptomatology, including constipation and diarrhea, may correlate with the severity of ASD [9,10].

Gut dysbiosis is also implicated in initiating systemic inflammation and neurological dysfunction [11,12]. Clinically, this bidirectional inflammation has been linked to dysregulation of neurobiology and neuroimmunology, which parallels the pathogenesis of GI symptomology in patients with ASD [11,12,13,14]. Recent studies have indicated microbiome-induced inflammation’s potential to alter the blood-brain barrier (BBB) permeability [5,15,16,17,18]. This review will discuss the predominant mediators of blood-brain barrier integrity and the signaling mechanisms by which they influence neurobiological development. A clear understanding of the neurobiochemical underpinnings will enable researchers to better understand aberrant neurodevelopmental mechanisms, such as in ASD. These mechanisms have significant implications for the development of novel therapies targeting specific dysfunctions in the GBA. Such therapeutics, if administered during critical periods of neurodevelopment, may have strong potential to minimize, prevent, and possibly reverse the neurocognitive deficits observed in ASD patients. We will discuss our current understanding of the gut microbiome and address the neurobiological impacts of gut dysregulation and inflammation. In particular, we will focus on the clinical relevance and contributory role of GBA dysregulation in aberrant ASD neurodevelopment along with future therapeutic prospects.

## 2. Gut-Brain-Axis

The gut-brain axis (GBA), also known as the microbiome-gut-brain axis, describes the complex bidirectional communication between the central nervous system (CNS) and enteric nervous system (ENS), which is highly modulated by gut microbiota (Figure 1). The multifaceted interactions of the GBA include linkage of peripheral intestinal functions with emotional and cognitive brain regions such as the hypothalamus, limbic system, and prefrontal cortex [19,20]. Physiologically, the GBA helps maintain gastrointestinal homeostasis and mediates neuro-immune-endocrine communication—notably, immune activation, intestinal permeability, entero-endocrine signaling, and enteric reflexes [21]. Recent advances in our understanding of the GBA have also demonstrated the contributory role of gut microbiota on motivation and higher cognitive functioning [11].

These bidirectional interactions occur via a complex integrated signaling pathway between the CNS, enteric nervous system (ENS), autonomic nervous system (ANS), and hypothalamic-pituitary-adrenal (HPA) axis [12]. The ANS, which is comprised of sympathetic and parasympathetic branches, regulates both afferent and efferent communication between the CNS and the intestinal tract [22]. The gut microbiome has been found to actively interact with the sympathetic nervous system extrinsic to the gut [23]. Recent experiments have shown that microbial depletion in the gut leads to increased expression of cFos, a marker of neuronal activity and circuits; colonization of germ-free (GF) mice with gut microflora suppressed this pathway via short-chain fatty acid (SCFA) action in the gut-associated sympathetic ganglia [24]. Moreover, it is also hypothesized that there is a more direct, bidirectional communication between the gut and the brain via vagal nerves. Retrograde neuronal tracing has identified links from the intestinal wall to specific brainstem nuclei, which are activated after microbial depletion, and has identified efferent links from sympathetic glutamatergic neurons in the brain which regulate GI function [24]. These pathways provide evidence for a functionally relevant bi-directional link between the gut and the brain. The brainstem nuclei are the nuclei in the brainstem that include cranial nerve nuclei, red nucleus, and substantia nigra.

The HPA axis predominantly oversees the body’s response to emotional or physiological stress [25]. Moreover, as an integral component of the limbic system, the HPA axis ultimately regulates the release of cortisol from the adrenal glands. Cortisol, the body’s primary stress hormone, plays a pivotal role in the systemic effects of the body’s adaptive response to stress, including effects on the gut microbiome [13]. Although the precise mechanism is not fully understood, cortisol is a potent regulator of immune function and has been demonstrated to induce pathologic shifts in gut microbiome composition and increase intestinal permeability [26,27]. Through parallel signaling pathways like these, reciprocal hormonal signaling between the CNS and ENS allows both the brain and endogenous gut microbiota to modulate enteric function [11].

## 3. Blood-Brain Barrier

**Figure 2:**
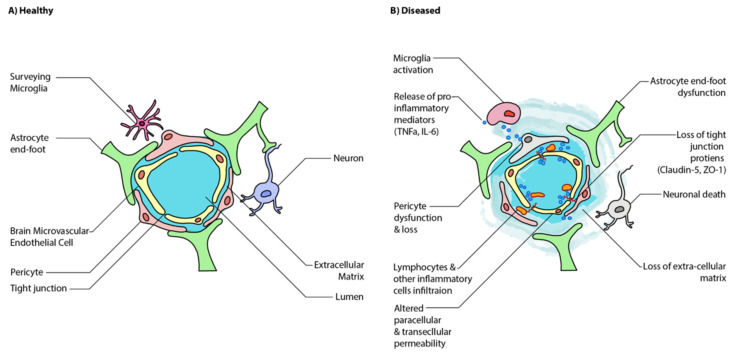
Schematic illustration of healthy and disease state blood-brain barrier (BBB) epithelium. (**A**) A healthy BBB is tightly sealed by the tight junctions of endothelial cells and astrocytes. (**B**) A disease state BBB has increased translocation of inflammatory mediators, immune cells, and microglial activation, which further increases BBB permeability. Ultimately, this results in neuroinflammation and neuronal cell death. Adapted from [5].

While the neuronal interplay between GBA is increasingly established, the gut may also affect the BBB’s integrity and alter functioning in the CNS itself. The BBB is formed by the tightly packed endothelial lining of the capillaries that supply the brain (Figure 2). It is designed to protect the brain from pathogens and to maintain homeostasis by preventing substances from diffusing freely into the brain. Passage across this barrier is determined by solubility across the lipid bilayer and recognition by specific transport molecules. The proteins sealing the gaps between endothelial cells are critical to this strict regulation, namely claudin-5,-11,-12,-25, and zonulin-1 (Figure 2) [28,29]. Interestingly, these same molecules are found in the intestinal epithelial barrier, where they mediate intestinal permeability [30]. Under disease state, BBB has increased translocation of inflammatory mediators, immune cells, and microglial activation, which further increases BBB permeability (Figure 2). These findings have been observed in human ASD brain samples where there is increased neuroinflammation coupled with an alteration in the expression of genes associated with BBB integrity and a corresponding decrease in gut barrier integrity [31].

The body’s immune system is critical for the proper development of the BBB. For example, the maternal gut microbiota plays a role in regulating the fetal BBB in the womb by upregulating the expression of proteins like claudin-5 [15]. This is an especially interesting finding considering many population-based studies have found an increased risk associated with maternal antibiotic use in the third trimester, particularly with penicillin [32,33]. This is supported by a murine study that found that low-dose penicillin in late pregnancy and early postnatal life increased long-term adverse effects in the offspring of the mice by increasing cytokine expression in the frontal cortex, modifying the blood-brain barrier integrity, and altering behavior [34].

Viral infections, often implicated in the pathogenesis of ASD, have also been associated with altering the BBB permeability. For example, HSV, HTLV-1, Rabies virus, West Nile virus, and Lymphocytic choriomeningitis virus (LCMV) are known to gain access to the brain. However, for many viruses, the precise molecular mechanisms underlying their entry and the extent of BBB disruption are unknown. Nevertheless, the principles behind viral entry and disruption of the BBB may be similar. The most studied virus in this regard is HIV-1. HIV-1 induces pathways that increase reactive oxygen species (ROS), upregulate inflammatory cytokines, proteasomal degradation of tight junction proteins within cells, and increases expression of matrix metallopeptidases (MMPs), which are key mediators of BBB dysfunction that act by degrading the extracellular matrix (ECM) and other protein-based molecules [35]. Furthermore, many viruses disrupt the actin cytoskeleton, which is essential for tight junction and BBB function [36]. In addition to disruption mediated directly in the barrier cells, viruses like rabies and LCMV induce CD8^+^ and CD4^+^ T cell dependent permeability [37,38]. Indeed, Epstein-Barr virus and Herpes Simplex virus 2, viruses identified as risk factors for ASD, can directly infect human brain endothelial cells [39,40]. Finally, it is worth mentioning that besides viruses and bacteria, many well-known parasites breach the BBB through similar mechanisms, including *Toxoplasma gondii* and *Plasmodium* spp., although associations have not been found between *Toxoplasma gondii* antibodies and ASD [35,39].

Regardless of the virus and its mechanisms, as the virus promotes inflammation and disrupts tight junctions, it allows cytokines, leukocytes like monocytes, macrophages, and T cells to enter the brain and accelerate disease progression [28]. Even if the viruses do not enter the brain, they can still activate an immune response from the microglia that may initiate neuroinflammation or encephalitis [41,42,43].

Additionally, it is interesting to note that endothelial and epithelial tight junctions have many similar features and share many of the same core claudins and occludins with varying degrees of permeability and are subject to the same mechanisms of disruption. This deepens the gut-brain connection by broaching the concept that common viral gastroenterophathies might also induce subclinical BBB permeability.

## 4. Gut-Brain Connection

Studies have demonstrated the importance of a functional gut microbiome in maintaining the integrity of the BBB. A recent study revealed that germ-free mice had increased BBB permeability compared with specific pathogen-free mice containing a healthy microbiota [15]. It has also been shown that the manipulation of the microbiota via antibiotic-treated or germ-free (GF) adult mice alter the gene expression and phenotype of microglia and neuronal cells, producing immature microglia reminiscent of developing juvenile cells as well as significantly altering gene expression in prefrontal cortical neurons, glia, and other cell types. Combined, these findings are hypothesized to be causal in the reduction of fear extinction learning associated with GF and antibiotic-treated mice. Briefly, fear extinction learning is the decline in a conditioned fear response following non-reinforced exposure to a feared conditioned stimulus, making it a measure of anxiety and emotional memory processing. Moreover, these findings were reversed upon the reestablishment of a functional microbiome during a crucial developmental window [44]. This supports the gut’s functional role in permanently shaping neurological functioning,

Another study provided additional evidence supporting the importance of the microbiome in neurodevelopment [45]. It was observed that after receiving microbiota transfer therapy (MTT), GF mice showed increased hippocampal neurogenesis and increased intestinal growth; further reinforcing the physiologic connection between the gut and brain. Additionally, metagenomic sequencing indicated enrichment in butyrate-producing microbes after MTT, implicating bacteria byproducts in the GBA homeostasis. The higher concentrations of gut-derived butyrate coincided with increased AMPK, SIRT-1 activation, and reduced mTOR signaling, which are critical mediators in energy regulation, the anti-inflammatory response, and autophagy, respectively [45]. These findings were further confirmed with exogenous administration of butyrate.

While evidence accumulates regarding the effect of the gut microbiome on brain development in general, similar findings have also been implicated in the pathophysiology of ASD. It was demonstrated that there is an altered expression of genes associated with both increased intestinal permeability and BBB integrity, coupled with increased neuroinflammation in ASD [31]. Further animal model studies have shown long-term changes in BBB permeability and white matter, following prolonged systemic inflammation in early development [46].

The cognitive effects of ASD can also be understood via the gut-brain axis association with the autonomic nervous system (ANS). A recent study by Kong et al. found that measures of autonomic function, gut microbiome markers, and autism behaviors, assessed by the Autism Treatment Evaluation Checklist (ATEC), were significantly associated with each other [47]. Specifically, alpha diversity was negatively correlated with ATEC total score, as well as the Sensory/Cognitive Awareness and Speech/Language subsections (higher ATEC scores indicate more severe symptoms). Additionally, other autonomic function markers such as body temperature and blood volume pulse (BVP) were significantly associated with ATEC scores. Moreover, these same autonomic indices correlated with changes in the gut microbiome, for example, a positive correlation between BVP and *firmicutes*/*bacteroidetes* ratio in all subjects. Importantly, although this study attempted to develop predictive models using these indices and ATEC scores, their models were not statistically significant [47].

Many specific diets have also been frequently hypothesized to play a beneficial role in improving cognition in individuals with ASD. For example, iron deficiency has been implicated in ASD’s adverse cognitive effects, given that it is frequently deficient in those with ASD and its importance in motor, behavioral, and cognitive development [48]. Moreover, it has been seen that ketogenic diets, gluten-free and casein-free diets, as well as increased intake of folic acid and vitamin D are beneficial in improving social, communicative, cognitive, and motor skills in children with ASD. However, it is vital to note that some of these studies contradict each other and refute their findings [49]. Moreover, it does not appear that any of these interventions are decisive factors in the development or progression of ASD. Rather, they positively contribute to beneficial trends within disease development, progression, and therapy. Future studies should make active efforts to design larger, randomized, blinded, and controlled trials to delineate the precise mechanisms of these diet’s effect [49].

## 5. Gut-Induced Inflammation and Altered Neuroimmunology

There are many causes of systemic inflammation found throughout the body. However, in the context of ASD, inflammation originating in the intestinal lumen is particularly of interest, as ASD manifests itself in early childhood and is often accompanied by GI distress. Throughout development, many factors can either be neuro-protective or neuro-harmful, including genetics, age, gender, diet, environmental exposures, drugs, and the microbiome [50]. Of all of the factors currently thought to be implicated in the pathogenesis of ASD neuroinflammation, the microbiome, via the GBA, offers promising avenues for therapeutic interventions.

Given that gastrointestinal (GI) comorbidities characterize ASD, it is not surprising that recent studies have implicated gut dysbiosis with the pathogenesis of ASD. ASD is a neurodevelopmental disorder with symptoms resulting from abnormal maturation of various brain systems [51]. The gut-brain communication can be direct or indirect. Directly, the central nervous system (CNS) predominantly interacts with the enteric nervous system (ENS) via the vagus nerve and other neural and endocrine connections. For example, enteroendocrine cells (EECs) produce gut hormones such as cholecystokinin (CCK) and glucagon-like peptide 1 (GLP-1) in response to intestinal stimuli that ultimately modulate ENS activity. In turn, ENS neurons synapse onto EECs, allowing for mutual feedback [52,53]. Indirectly, however, the gut interacts with the brain via a multitude of intermediates, including microbial metabolites, virulence factors, and cytokines, among many others. For this to occur, these intermediates must cross through two evolutionarily linked barriers, the intestinal epithelium and the blood-brain barrier (BBB).

Interestingly, it appears that the same factors affecting gut permeability also affect the permeability of the BBB. These disruptions in barrier integrity open the way for inflammation. Increased permeability of the intestinal epithelium facilitates the translocation of intestinal components such as gram-negative bacteria and lipopolysaccharide (LPS) from the lumen to the mesenteric lymph and peripheral circulation [54]. Indeed, damaged, inflamed, and therefore permeable epithelia are the main routes utilized by commensal bacteria to migrate to the bloodstream [55]. In turn, the translocation of pro-inflammatory molecules across the intestinal barrier causes a low-grade systemic inflammatory response, which can alter the BBB’s permeability [28,56].

The initial intestinal epithelial cell damage arises due to a dysbiotic gut microbiome and the consequences of an altered metabolomic profile. For example, the standard western diet and certain mucolytic bacteria can cause a thinning of the protective mucus barrier lining the intestinal epithelium. This mucous membrane degradation puts the gut lumen’s contents in direct contact with the intestinal epithelium allowing for increased translocation of pro-inflammatory molecules like LPS and even bacteria themselves [55,57]. Once in the systemic circulation, these factors initiate a diffuse low-grade inflammation similar to the type that increases the BBB’s permeability [28,56]. Based on these examples, it is plausible to link the dysbiosis associated with ASD in developing systemic and neuroinflammation through the dysregulation of these reciprocal interactions found in homeostatic, healthy tissue.

The microbiome’s tangible effects on systemic and neuroinflammation are likely to affect lymphoid cells and the adaptive immune response itself directly. In general, microbial components are known to interact directly with toll-like receptors (TLRs), antigen-presenting cells, differentiated T cells, CD4 T cells, and B cells [58]. Specifically, experiments with GF mice had a decreased proportion of T cells and B cells in the gut, while other cell populations were unaffected [59]. Another study found that prenatal microbiome composition was critical in developing behavioral abnormalities in a murine maternal immune activation (MIA) model of autism. It was demonstrated that pre-conception MTT can transfer vulnerability to MIA-associated neurodevelopmental disorder and that this is linked to alteration of the maternal immune system. Crucially, as we will discuss further, T_H_-17 cells were implicated in the mechanism as IL-17a ablation protected neurodevelopmental abnormalities in ASD offspring [60,61]. Additional studies have reinforced this finding by implicating gut induced inflammation in T_H_-17 activation and T_H_-17 lymphocytes in increasing systemic inflammation and promoting BBB disruption and CNS inflammation [62,63,64,65]. Other studies have subsequently tied the digestive tract T_H_-17 to induced systemic inflammation.

These findings suggest how the microbial landscape can directly moderate immune-related neurodevelopmental diseases, including ASD. However, the microbial metabolites that comprise the metabolome are likely the most significant contributors to systemic inflammation and subsequent neuroinflammation.

### 5.1. Gut Microbiota-Derived Metabolites

Of the numerous pathways for the gut to induce neuroinflammation, microbial metabolites play a crucial role, especially the short-chain fatty acid (SCFA) butyrate [66]. Decreases in butyrate can lead to generalized inflammation through a variety of mechanisms, including increased NF-κB synthesis and LPS absorption; this, in turn, can increase the permeability of the blood-brain barrier, thereby opening the way for neuroinflammation [67].

Beginning in the gut, SCFAs are the byproducts of microbial fermentation of prebiotics. Butyrogenic bacteria span phyla and classes; however, a few key groups have been pinpointed as key in butyrate production, including *Bifidobacteria*, which are decreased in regressive autism, and the *Clostridia* class of *Firmicutes* [68,69]. Interestingly, it appears as if no single strain is responsible for SCFA production; instead, they are the byproducts of complex cross-feeding pathways. This concept was supported in recent studies showing that in GF mice, only supplementation with multiple probiotic species stimulated protective mucin production, this pathway is stimulated by SCFA production likely via free fatty acids (FFAs) [21,57].

SCFAs serve a vital role in the gut as the preferred energy source for the intestinal epithelium, especially colonocytes, via the β-oxidation pathway [69]. Additionally, butyrate can directly alter gene transcription by functioning as an inhibitor of histone deacetylases (HDACs), promoting histone acetylation and gene expression stimulation in host cells. In this capacity, SCFAs have been shown to strengthen the intestinal barrier by upregulating and reorganizing tight junctions connecting epithelial cells [69].

Outside of the gut, the SCFAs receptors are found throughout the body on transmembrane proteins and transporters and are expressed by a variety of cells, including neurons, implying their ubiquitous physiologic functions [70]. There is accumulating evidence showing that butyrate has significant anti-inflammatory effects via the induction of regulatory T cells (T_reg_) [71]. Butyrate also was found to suppress gene expression and LPS-induced secretion of several pro-inflammatory genes in endothelial cells [72]. However, it is essential to keep in mind that fecal SCFA concentrations are not always representative of serum concentrations or even colonic concentrations [69]. Bourassa et al. speculate that a high fiber diet increases systemic butyrate from the distal colon blood supply, which bypasses the portal vein; this allows butyrate to have a significant impact on brain function and inflammation via its role in regulating the BBB integrity [73].

This systemic inflammation mediated by the metabolome and butyrate, in turn, affects the brain function. Directly, butyrate has been shown to exert potent neuro-pharmacological effects via the vagus nerve, altering social communication [69]. Furthermore, studies have shown that butyrate could exert specific neuroprotective effects by decreasing TNF-α, IL-1β, and IL-6 in the brain [74,75]. Additionally, butyrate enhances inhibitory signaling in the mouse models of autism even at relatively low doses (100 mg/kg), which, although not inducing significant differences in histone acetylation in the prefrontal cortex, still attenuated social deficits [76]. This is particularly interesting, as ASD is characterized by decreased neuro-inhibitory function.

### 5.2. Lymphatics

The lymphatic system is responsible for disposing of toxins, wastes, and pathogens. It consists of lymphatic vessels connected to lymph nodes found through the body and include structures like the tonsils, adenoids, spleen, and thymus. This system is crucial for coordinating the body’s immune response to infections.

Recently, a study found a direct connection between the brain and the lymphatic vessel network in the meningeal linings of the brain, which drain into the deep cervical lymph nodes [77]. Consequently, brain lymphatic drainage plays an essential role in maintaining homeostasis by regulating water and ion balance, waste clearance, and reabsorption of solutes and coordinating the immune surveillance and responses of the brain [78]. Moreover, the gut is directly connected to the lymphatic system via lacteals, which transport chylomicrons directly from intestinal epithelial cells to the thoracic duct and systemic circulation [28,79,80]. This connection means that lymphatic vessels could act as direct communication between the brain and the peripheral immune system, making it yet another factor connecting the brain and the immune system. Given the hypothesized connection between early gut-induced inflammation, increased BBB disruption, and neuroinflammation, this gut-lymphatic connection is increasingly important.

Additionally, adequate lymphatic function is vital for intestinal function and structure. A recent study has shown that lacteal function in the gut is a crucial regulator of angiogenesis in the intestine. It was found that the ablation of intestinal lacteals disrupted blood vessel and villous architecture, which lead to increased invasion of intestinal pathogens into the circulatory system [80]. Indeed, common disease states like IBS and IBD have been shown to reduce the functionality of lymphatic vessels as well [81,82]. As discussed previously, this, therefore, may induce systemic inflammation and subsequent neuroinflammation. Given the hypothesis that early gut induced inflammation leads to BBB disruption, the lymphatic system likely takes an increasingly central role concerning the GBA because of its role in regulating the inflammatory response. However, knowledge of the connection between the brain and the lymphatic system is still very recent, and more clinical research is needed to understand the precise role the lymphatic system plays in the onset of neurological disease, particularly in ASD.

### 5.3. Microglia

Another element connecting the immune system with the microbiome is the microglia. Microglia are integral regulators of neuro-inflammatory and CNS immune responses to insults. Subsequent inflammation is partially regulated by gut microbiota; however, this synergistic inflammatory relationship has yet to be thoroughly investigated.

In addition to their role as the macrophages of the brain, microglia are also key players in early brain development. Interestingly, microglia during early development are wholly different from mature microglia present later in life [83]. Recent experiments have shown that the gut microbiota contributes to the maturation of naïve microglia and, in the absence of microbiota, the number of mature microglia decreases while the total count of microglia remains the same. However, understanding the mechanisms that regulate the maturation and function of microglia in vivo is limited [84]. Moreover, another study demonstrated that host microbiota continuously controls maturation and function of microglia in the CNS, since temporal eradication of host-microbiota severely changed microglia properties, while recolonization with a complex microbiota partially restored microglial function [85].

It is well known that microglial dysfunction is a major contributor to the initiation and progression of many neurological diseases such as multiple sclerosis, Alzheimer’s disease (AD), depression, Parkinson’s disease, and autism [84]. For example, in AD models, activated microglia produced excess pro-inflammatory cytokines like IL-1β and TNF-α that ultimately induce neurodegeneration and further exacerbate the pathological processes of AD. Interestingly, supplementation with *Clostridium butyricum* significantly reduced cognitive deficits, microglia activation, neurodegeneration, and Aβ deposition while increasing butyrate levels [75]. These mechanisms are particularly relevant for ASD as autism is a neurodevelopmental disorder beginning in early childhood, a time when the brain is especially vulnerable [86]. This makes the microbiota’s role in the maturation of the microglia particularly relevant.

Systemic inflammation via translocation of immunogenic molecules and pathogens across the intestinal epithelium and the compromised BBB initiates an inflammatory response in the brain. In ASD, postmortem tissue and animal models have indicated that there are increased numbers of reactive microglia and astrocytes in neural tissues [87]. Although their precise role remains unclear, it is hypothesized that the inflammatory cells alter neuronal connectivity and interfere with cellular communication.

These factors are further modified by intestinal dysbiosis. Mouse studies have shown that gut microbes produce compounds that prime cells to destroy harmful viruses in the brain and nervous system. Mice treated with antibiotics that disturbed the gut microbiome’s ecology before the onset of disease were left defenseless since they had less microglia that would be flagging the viruses for removal by the immune system [88]. This demonstrates how gut immune-stimulatory factors could influence microglia function to prevent CNS damage following viral infection.

## 6. The Impact of Gut Dysbiosis in Early Childhood on Neurodevelopment

During early childhood, the brain is particularly predisposed to remodel synaptic connections and neuronal circuits in response to internal and external stimuli. Critical periods of early neurodevelopmental are characterized by extreme plasticity in neuronal circuitry, which allows for efficient remodeling during the postnatal period. However, anomalous stimuli encountered during these critical periods are more likely to prompt altered neurodevelopmental trajectories [89]. Notably, gut microbiota’s maturation closely parallels postnatal brain development, and recent studies have demonstrated the influential role of early gut microbial signaling regarding neurophysiological functional development [90]. While the exact neurobiochemical signaling mechanisms are still being investigated, there is considerable evidence to support that postnatal gut flora has a remarkable propensity to influence neurodevelopment and behavioral outcomes [74,78,91].

ASD is heterogenous in onset, with most children exhibiting symptoms of social and developmental regression around the age of two years old [92]. Interestingly, the microbiome quickly matures during the first few years of life and undergoes a dramatic maturation to resemble an adult gut ecosystem [93]. In vivo studies of germ-free (GF) mice—sterile animals with no microorganism contact—demonstrate impaired social functioning and excessive self-grooming behavior, which parallels repetitive stereotactic behaviors in children with ASD. Subsequent colonization of GF mice partially reversed the phenotype by stabilizing the social avoidance and self-grooming behavior; however, social cognition deficits persisted [94]. This signifies that the critical periods for gut microbial influence on neural circuitry may be distinct for the development of social, behavioral, cognitive, and sensory modalities.

Additionally, maternal microbial health is implicated in fetal microbiome composition and postnatal developmental trajectories [89]. Perinatal antibiotic use in mice models demonstrated a marked disruption in the microbiome of both the mother and offspring, followed by an observed decrease in pup exploratory activity at four weeks of age [95]. Another in vivo study of perinatal antibiotic exposure in rats noted the development of poor social interactions, anxiety, and pre-pulse inhibition, commonly associated with behavioral health disorders, including ASD and schizophrenia [96]. While several recent studies corroborate these observed trends, there is ultimately no consensus regarding the risk of antibiotic use during pregnancy on neurodevelopment due to the lack of specific mechanistic understanding.

Commensal gut bacteria have a remarkable ability to influence the development of neuronal function and behavior. In particular, the possibility of critical periods for gut microbiome development is important to consider in our understanding of the mechanistic modulations that occur during these periods of plasticity. Once these molecular signaling pathways are further delineated, there is strong potential for targeted microbiota-derived therapeutics to treat neurodevelopmental diseases.

## 7. Limitations

While many of the studies discussed above are very promising, they are not without their limitations, this review included. Foremost, many studies rely on various experimental models to test their hypotheses and interventions limiting their external validity. For example, murine models were frequently used to examine the effect of the gut microbiota, which has obvious limitations in extending its conclusions to humans [15,24,34,44,45,59,88,94]. Additionally, many experiments, particularly those analyzing immune cell function, are carried out in vitro; this has proven particularly challenging in extrapolating in vitro interventions to humans. For example, while various butyrate concentrations can modify immune cell differentiation and reactivity in vitro, similar changes have not always been seen in human experiments [45,71]. This leap in experimental complexity remains a challenge to be addressed in future studies. Nevertheless, despite these limitations, observed morphological and behavioral changes in these model systems still serve as valuable starting points for future hypothesis induced investigations in human trials.

Additionally, while this is not a systematic review of each study’s limitations, a few characteristic studies are worth noting. Many of the studies were limited in the sample size. Kang et al. contained only 18 participants and had an open-label design, meaning that it was not placebo-controlled, blinded, or randomized. This is a significant limitation, as many of the behavioral tests used are prone to the placebo effect. Additionally, they lacked many important exclusion criteria, including functional food intake (e.g., fiber) and dietary supplement use. However, many of the subjects’ symptoms continued to improve even after two years of follow up, thus decreasing the concern over the placebo effect [8].

Adams et al. had a larger, albeit still modest sample size for such a heterogeneous population, containing 58 children with ASD and 39 controls. However, this study failed to stratify participants by ASD severity, which could affect response to treatment. Finally, Adams et al. was one of the many studies utilizing stool culturing techniques to analyze the gut microbiome instead of next-generation sequencing tools. Stool culturing significantly limits the description of the gut microbiome as many species are not easily culturable [10].

In the present study, the investigations included were limited to papers searchable on MEDLINE-PubMed, Science Direct, Web of Science, and Scopus databases. This means that non-English language, unpublished studies, or studies not archived on the searched databases were not included in the review and may skew the results towards more positive results. Additionally, some of the hypotheses put forth in this article are based on mechanisms and processes seen in other disease processes such as IBD or multiple sclerosis and may not hold true for ASD. The conclusions of this study are also limited by the heterogeneous nature of the studies analyzed. Most of them exhibit different experimental designs, varying strengths and types of interventions, and levels of control at baseline.

## 8. Conclusions

The ability of the microbiome to alter BBB permeability has been of particular interest within the context of neurodevelopment and the onset of ASD. Compositional alterations in the gut microbiome and its metabolome may be an underlying source for systemic inflammation and, ultimately, neuroinflammation. However, the molecular mechanisms that underlie microbiota-induced alternations in postnatal neurophysiology are not yet well understood.

The BBB is essential for maintaining the immunological integrity of the CNS to prevent neuroinflammation. Dysregulation of the gut’s protective mucous membrane provides a conduit for pro-inflammatory molecules, such as LPS, and bacteria to translocate from the intestinal epithelium into the gut lumen. The subsequent inflammatory response is a result of the dysfunction of key regulatory molecules that maintain not only intestinal integrity but also the BBB integrity.

Recent research has revealed a direct connection between the brain, the meningeal lymphatic vessel network, and the gut lymphatic system, which may serve as a bridge between the central and peripheral immune systems. Another element connecting the immune system functioning to the microbiome are microglia, which are integral regulators of neuroinflammation. In fact, it has been demonstrated that the maturation of naïve microglia is in great part controlled by the microbiome. Moreover, in addition to affecting microglial maturation, the host-microbiota has also been shown to modulate glial activation as well, thereby deepening the microbiome’s role in regulating neuroinflammation in the CNS.

As the prevalence of ASD and other neurodevelopmental disorders continues to rise, increased neuro-biochemical advances that focus on the GBA will provide clinically relevant targeted biotherapies. The implications of these advances and biotherapies can be profound given that many children and infants are frequently prescribed and overprescribed multiple doses of antibiotics that can disturb the development of their microbiome, leading to an inflammatory state at critical stages of brain development without being detected.

There is still much to be discovered in terms of understanding the bidirectional communication network between the CNS and the microbiome ecosystem. The future direction holds exciting potential to reveal future avenues of microbiota-based therapeutics to treat neurodevelopmental disorders. Further studies are warranted to delineate the specific molecular signaling pathways implicated in neurodevelopment, with the strong potential to catalyze targeted microbiota-derived therapeutics.
